# Surveillance Mammography Behaviors in Black and White Breast Cancer Survivors: Behavioral Risk Factors and Surveillance System, 2016

**DOI:** 10.1186/s12905-023-02246-x

**Published:** 2023-03-30

**Authors:** Megan C. Edmonds, Bassam Dahman, Sunny Jung Kim, Kandace P. McGuire, Vanessa B. Sheppard

**Affiliations:** 1 Division of General Internal Medicine, Icahn School of Medicine at Mount Sinai, 17 E. 102 St, New York, NY USA; 2 Department of Health Behavior and Policy, School of Medicine, Virginia Commonwealth University, Richmond, VA USA; 3 VCU Massey Cancer Center, Richmond, VA USA; 4 Department of Surgery, VCU Massey Cancer Center, Richmond, VA USA

**Keywords:** Surveillance mammography, Breast cancer survivors, Socioeconomic status, Health disparities

## Abstract

**Background:**

Surveillance mammography is recommended annually for early detection of disease relapse among breast cancer survivors; yet Black women have poorer national rates of surveillance mammography compared to White women. Factors that influence racial disparities in surveillance mammography rates are poorly understood. The purpose of this study is to evaluate the contribution of health care access, socioeconomic status, and perceived health status on adherence to surveillance mammography among breast cancer survivors.

**Methods:**

This is a secondary analysis of a cross-sectional survey among Black and White women ≥ 18 years, who reported a breast cancer diagnosis and completed breast surgery and adjuvant treatment from the 2016 Behavioral Risk Factor Surveillance System National Survey (BRFSS). Bivariate associations (chi-squared, *t*-test) for independent variables (e.g., health insurance, marital status) were analyzed with adherence to nationally recommended surveillance guidelines defined as two levels: adherent (received a mammogram in the last 12 months), vs. non- adherent (“received a mammogram in the last 2–5 years, 5 or more years or unsure). Multivariable logistic regression models were used to evaluate the relationship between study variables with adherence, while adjusting for potential confounders.

**Results:**

Of 963 breast cancer survivors, 91.7% were White women with an average age of 65. 71.7% reported a surveillance mammogram in the last 12 months, while 28.2% did not. Diagnosed > 5 years (*p* < 0.001); not having a routine checkup visit within 12 months (*p* = 0.045); and not seeing a doctor when needed due to cost (*p* = 0.026), were significantly related to survivor’s non-adherence to surveillance mammography guidelines. A significant interaction was found between race and residential area (*p* < 0.001). Compared to White women, Black women living in metropolitan/suburban residential areas were more likely to receive surveillance guidelines (OR:3.77;95% CI: 1.32–10.81); however Black women living in non-metropolitan areas were less likely to receive a surveillance mammogram compared to White women living in non-metropolitan areas (OR: 0.04; 95% CI: 0.00–0.50).

**Conclusion:**

Findings from our study further explain the impact of socioeconomic disparities on racial differences in the use of surveillance mammography among breast cancer survivors*.* Black women living in non-metropolitan counties are an important subgroup for future research and screening and navigation interventions.

## Introduction

**Fig. 1:**
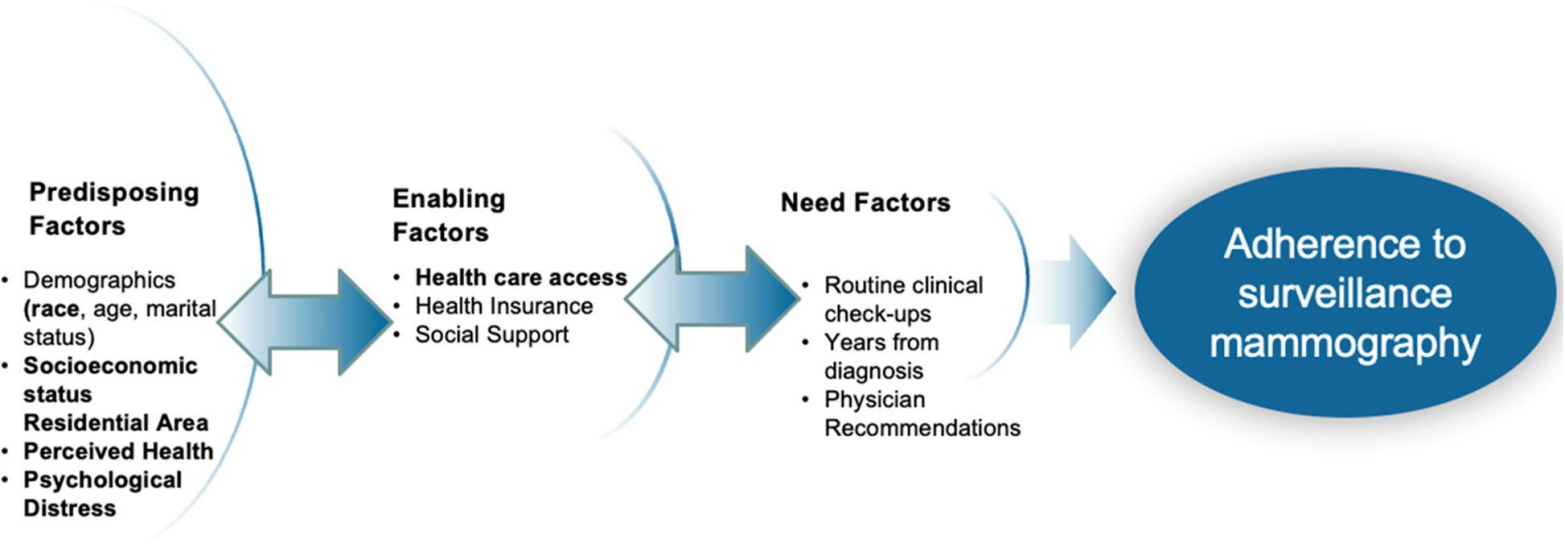
Behavioral Model For Vulnerable Populations

Adherence to surveillance mammography in women with residual breast tissue is a salient guideline recommended by the American Cancer Society for early detection [1]. Mammographic detection has shown clinical benefit for earlier stage detection of recurrent disease and a 39% decrease in mortality [2]. Unfortunately, evidence shows Black BC survivors are less frequently receiving surveillance mammography within the first five years compared to their White counterparts, with few explanations for these disparities [3–6]. Factors that influence adherence to surveillance mammography are complex and include socioeconomic status, employment, marital status, age and provider and system level factors (e.g., routine clinical visits) [4, 6, 7]. Most research however lack the use of a multifaced framework to fill gaps of psychosocial and residential factors that may also influence survivors adherence to surveillance mammography guidelines. In this study, we apply the Behavioral Model for Vulnerable Populations (Fig. 1) [8] that posit health care utilization is directly influenced by three domains: *predisposing (e.g., population characteristics), enabling (e.g., social resources) and need (e.g., perceived illness).*We use this conceptual framework to explicate a better understanding of surveillance behaviors among BC survivors-a vulnerable group to systemic racism, medical mistrust, perceived discrimination and disease relapse [9–11].

Studies observing screening mammography have found that lower socioeconomic status, residential area (*predisposing factor*) and health care access (*enabling factor*) are associated with not screening [12–15]; yet very few studies have observed these factors among BC survivor’s surveillance mammography behaviors [4, 16].Earlier studies using NCI’s Surveillance, Epidemiology, and End Results (SEER)-Medicare data reported that socioeconomic disadvantages in terms of SEER geographic regions, and having lower income were key determinants of non-adherence to surveillance mammography [4, 16]. For example, surveillance mammography was 30% lower for Black women versus White women who lived in areas with lower median income based on census tract of residence [7]. Another study found higher adherence was linked with having private or Medicare insurance when compared to public insurance [17]. Further examination is needed to determine whether racial differences exist in the associated role of health care access and residence composition with surveillance mammography, using robust national population-based datasets. Doing so will help to widen our understanding of socioeconomic disparities among survivors.

Changes in survivors’ physical and mental well-being [18, 19]may impact their surveillance behaviors, however there are limited studies in the surveillance setting that examine these psychosocial factors. Results from a large-population based study found young and uninsured Black BC survivors, with a lack of social support represented the poorest health-related quality of life profile at 25 months post diagnosis [20]. Similarly another study found higher levels of distress specific to breast cancer and mammography-related anxiety were associated with lower adherence to surveillance mammography [21]. Research is needed to further understand the nature of survivor’s well-being and the role of their perceived health influence on surveillance behaviors. This study will fill important knowledge gaps by assessing the relationship between perceived health outcomes with adherence to surveillance mammography.

The primary aims of this study are to: (1) evaluate whether White race (predisposing), increased health care access (enabling), lower levels of psychological distress and perceived health (enabling), will be associated with adherence to surveillance mammography; and (2) to assess if residing in a non-metropolitan county will be associated with non-adherence to surveillance mammography and differ by race [19, 22–24]. Guided by the Behavioral Model for Vulnerable Population, the aims of this study will fill scientific gaps in the surveillance setting (Fig. 1). This study will also be the first to retrospectively examine surveillance mammography among Black and White BC survivors, utilizing the 2016 Behavioral Risk Factor Surveillance System (BRFSS) nationally represented dataset.

## Methods

### Data source

Data from the 2016 Behavioral Risk Factor Surveillance System (BRFSS) was utilized in this study to construct a cohort of breast cancer survivors. BRFSS is the largest state-based representative telephone health survey in the US, with more than 500,000 respondents operated by public health departments, across 50 states and selected US territories, that utilizes a stratified design to collect landline samples [25, 26]. BRFSS is administered annually among 18 ≥ US adults to observe preventative health care practices (e.g., physical activity), chronic health conditions (e.g., diabetes), health risk behaviors (e.g., smoking) and demographic factors (e.g., age, race), which represents items from BRFSS core Section [27]. State-added questions and optional modules on specific health topics such as cancer survivorship, are available by request for state health departments to administer, using computer aided telephone interviewing [26]. The 2016 BRFSS was selected as an ideal survey year to observe trends of surveillance mammography among BC survivors, because this survey year measured personal history of breast cancer and mammography screening utilization [28].

### Study population

**Fig. 2:**
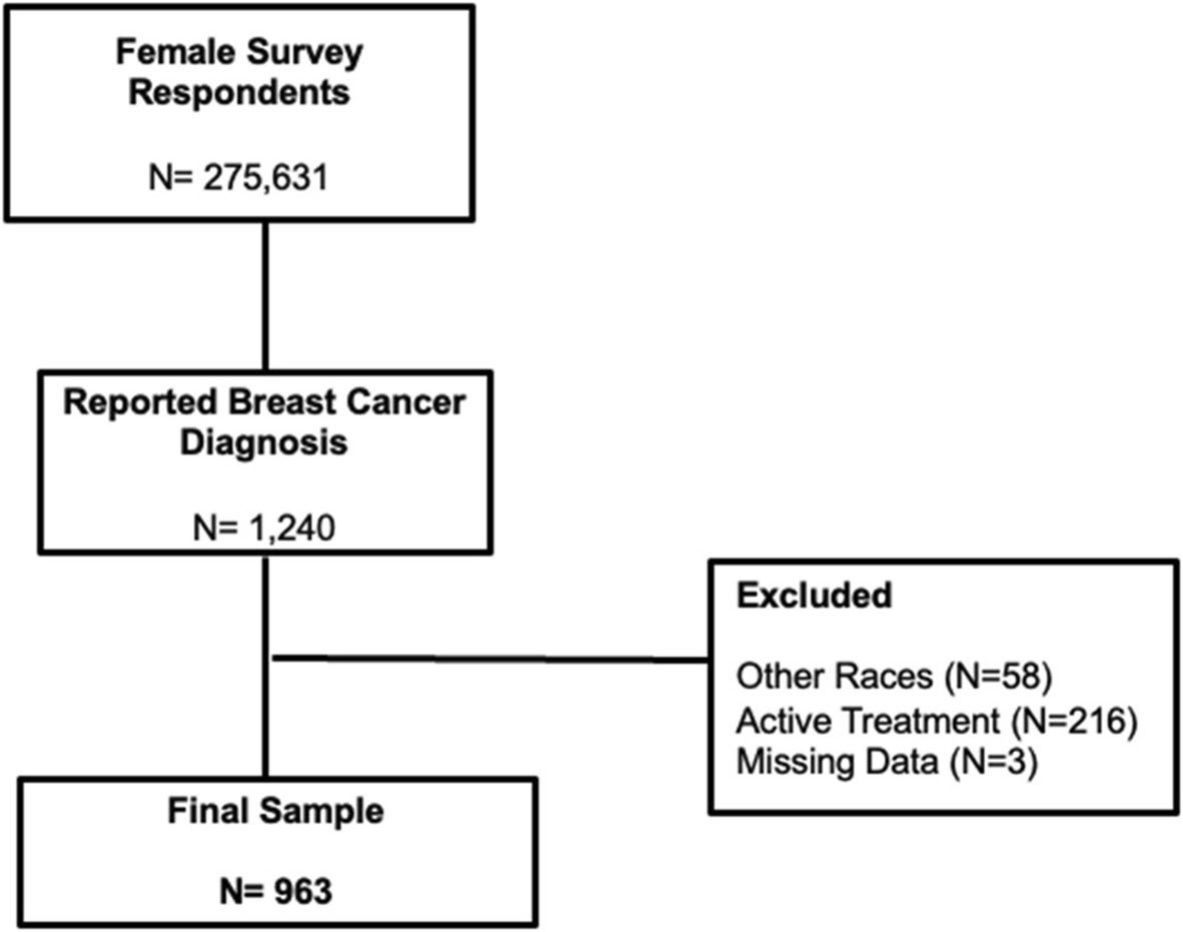
2016 BRFSS Breast Cancer Cohort Schema

This cross-sectional study design included a cohort of Black and White non-Hispanic female BC survivors (*N* = 963), who self-reported a diagnosis of breast cancer. To avoid a diagnostic mammogram at the time of the study participants were included in the analytic sample when meeting the following criteria: (1) Black non-Hispanic/or non-Hispanic White female, (2) reported a breast cancer diagnosis, and (3) completed breast cancer treatment such as surgery and adjuvant treatment. Exclusion criteria: missing values of their last mammogram, and race/ethnicity. The cohort procedure followed a similar schema of recently published studies of BC survivors (Fig. 2) [4, 29].

### Outcome variable

Adherence to surveillance mammography was determined using ASCO/ACS 2016 recommended screening guidelines of surveillance for breast cancer recurrence (defined as annual mammography screening) [1].Adherence was ascertained from the question “when was your last mammogram”. Survivors responses to this question were categorized into two levels: adherent to ASCO/ACS recommended guidelines (“received a mammogram in the last 12 months”), or non-adherent to ASCO/ACS recommended guidelines (“received a mammogram in the last 2–5 years, 5 or more years or unsure) [30].Our adherence definition is consistent with prior studies classification of mammography use among BC survivors [31, 32]. (Note there were only 3 women who were unsure).

### Independent variables

Predisposing Factors included age at the time of survey categorized as (< 50,50–65, ≥ 65) race was classified using race and ethnicity grouping (non-Hispanic White, non-Hispanic Black), and marital status (married, non-married). Socioeconomic status was measured using women’s metropolitan statistical area (MSA) categorized as residing in the center city of a MSA, inside a suburban county of a MSA and inside the county containing the center city compared to not residing in a metropolitan county [33, 34]. Perceived health status was evaluated with three survey items: (1) women’s overall perceived health, “Would you say that in general your health is?” Responses were categorized as “Very good”, “Good” and “Poor”; (2) women’s perceived psychological distress was measured from the question, “Now thinking about your mental health, which includes stress, depression, and problems with emotions, for how many days during the past 30 days was your mental health not good?”; and (3) women perceived physical or mental health “During the past 30 days, for about how many days did poor physical or mental health keep you from doing your usual activities, such as self-care, work, or recreation?” Respondents possible scores ranged from 0 (none of the past 30 days) to 30 (all of the past 30 days).

Enabling Factors included having a personal doctor from the question “What type of doctor provides the majority of your health care?” emotional support which was measured with the question “How often do you get the social support and emotional support you need? Scores were assessed on a 5-point Likert scale ranging from Always to Never. Respondent’s employment status at the time of interview was dichotomized as yes vs. no if they reported they were currently working vs. no. Health care access was measured if participants did or did not have access to health care due to cost, with two survey items, questions included: (1) “Was there a time in the past 12 months when you needed to see a doctor but could not because of cost?”; (2) “Were you ever denied health insurance or life insurance coverage because of your cancer?” Responses were reported as yes/no. Health care insurance was measured by asking participants if they had any form of health care coverage, with a yes/no response.

Need Factors categorized years from cancer diagnosis as (≤ 5 and > 5 years). Time elapsed from cancer diagnosis was based on NCCN enhanced surveillance definition for the first 5 years [35]. Women’s receipt of provider recommendation for surveillance routine check-up, “Have you EVER received instructions from a doctor, nurse, or other health professional about where you should return or who you should see for routine cancer check-ups after completing treatment for cancer? with a yes/no response. Women’s routine check-up was defined by 2016 ASCO/ACS recommended clinical follow-up guidelines (defined as annual follow-up visits), using the survey question, “About how long has it been since you last visited a doctor for a routine checkup?” Responses were categorized into two categories: adherent (“visited a doctor in the last 12 months”), vs. non-adherent (“visited a doctor in the last 2–5 years or 5 or more years”) [1].

## Data analysis

All data management procedures were weighted to determine national estimates of study variables and unbiased standard errors. Detailed methods about BRFSS sample weighting to account for the complex sample design are described elsewhere [36]. Descriptive statistics were employed to characterize the study cohort. Bivariate analyses χ^2^ and *t*-test were used to test the associations between categorical and continuous independent variables on the binary outcome: adherent to ASCO/ACS recommended guidelines (received mammogram in the last 12 months), versus non-adherent to ASCO/ACS recommended guidelines (received a mammogram in the last 2–5 years or none at all). For adjusted analyses, we used binary logistic regression to assess the association between predisposing, enabling and need predictors with surveillance mammography adherence. To assess whether race (non-Hispanic White = 1, non-Hispanic Black = 2) modified the effect of metropolitan residential area (Metropolitan/Suburban County = 1, Non-Metropolitan County = 2) on surveillance mammography within 12 months, we compared logistic regression models with and without an interaction term between race and metropolitan status. A significant interaction was found between race and metropolitan status on adherence to surveillance mammography *p*< 0.01, thus a race-residence composite variable was created in subsequent analyses. The multivariable logistic regression model was adjusted for predisposing (e.g., race, age, marital status) enabling (e.g., health insurance) and need (e.g., comorbidities, BC stage) factors, consistent with prior research assessing BC screening utilization among BC survivors [4, 37, 38]. Mean and mode imputation methods [39]were used to handle missing data on the following study variables: metropolitan status, age, employment, routine check-up, received follow-up instructions, denied health insurance, and poor health. All statistical analyses were conducted using SAS Version 9. (Due to missing cells for reporting low to no emotional support in the study sample, emotional support was removed from all final analyses.

## Results

Nine hundred sixty-three BC survivors with a mean age of 66 were included in our study sample. 91.7% (*n* = 884) were White and 8.2% were Black (*n*= 79). A majority had health insurance (98%), were married (49%), and lived in metropolitan/suburban counties (80%). 71.7% of BC survivors reported that they received a surveillance mammogram within the last 12 months compared to 28.2% who reported they did not; this finding is consistent with other reports [40]. The distributions of adhering to surveillance guidelines in predisposing, enabling and need model factors are presented in Table 1. While there were no significant differences in predisposing factors on adherence; it’s important to note that higher levels of psychological distress were among adherent survivors (mean = 64.2) compared to survivors who were non-adherent (mean = 58.8). For enabling predictors, women who saw a doctor without cost as a barrier were significantly more adherent to surveillance (95.8%) compared to women who could not see a doctor when needed due to cost (4%) (*p* = 0.026). For need factors, women who had their last routine checkup (e.g., physical exam) within 12 months at the time of interview (90%) were more adherent vs. women reporting their last routine checkup within 2–5 years (9.9%) (*p* = 0.045). Survivors diagnosed > 5 years (75%) were found to be less adherent compared to women diagnosed ≤ 5 years (24%) (*p* < 0.001).

**Table 1:** Characteristics of the 2016 BRFSS non-Hispanic Black and non-Hispanic White BC Survivors *N* = 963

	**Adherence to Surveillance Mammography Guidelines**		
	Yes	No	
	No (%)	No (%)	*p*-value
	691(71.7%)	272(28.2%)	
**Predisposing Factors**			
*Age*			
< 50	25 (3.6)	15 (5.5)	
50–64	170 (24.6)	72 (26.4)	0.811
≥ 65	496 (71.7)	185(68.0)	
Race			
non-Hispanic Black	64 (9.2)	15 (5.5)	0.509
non-Hispanic White	627 (90.7)	257(94.4)	
Marital Status			
Married	351(50.7)	123 (45.2)	0.749
Not married	336 (48.6)	148 (54.4)	
Residence			
Metropolitan County	553(80.0)	208 (76.4)	0.470
Non-Metropolitan County	138 (19.9)	64 (23.5)	
Perceived Health			
Good	541 (78.2)	204(75)	
Fair	107 (15.4)	46(16.9)	0.654
Poor	42 (6.0)	22(8.0)	
Physical Health (M + SD)	58.04 ± 2.0	55.13 ± 3.5	0.479
Poor Health (M + SD)	57.73 ± 1.6	57.09 + 2.5	0.833
Psychological Distress (M + SD)	64.20 ± 1.9	58.81 ± 3.5	0.172
**Enabling Factors**			
Health Insurance			
Yes	684 (98.9)	268 (98.5)	0.697
No	7 (1.0)	4 (1.4)	
Employment Status			
Employed	150 (21.7)	54 (19.8)	0.311
Non-employed	541 (78.2)	218 (80.1)	
Could not see doctor because of cost			
Yes	28 (4.0)	20 (7.3)	0.026*
No	662 (95.8)	250(91.9)	
Denied health insurance due to cancer			
Yes	58 (8.3)	26(9.5)	0.178
No	633(91.6)	246(90.4)	
Have a personal doctor			
Yes	669 (96.8)	254 (93.3)	0.374
No	22 (3.1)	17 (6.28)	
**Need Factors**			
Survivor Years			
≤ 5	244 (35.3)	66(24.2)	0.001*
> 5	447(64.6)	206 (75.7)	
Received Follow-up Instructions			
Yes	565 (81.7)	206 (75.7)	0.346
No	126 (18.2)	66 (24.2)	
Length of time since last routine checkup			
Within 12 months	622 (90.0)	237 (87.1)	0.045*
Within 2–5 years	69 (9.9)	35 (12.8)	

^*^ *p* < 0.05; **p < 0.01

## Multivariable analysis

The multivariable logistic regression model assessed the association between predisposing (e.g., *residential area*), enabling (e.g., *health care access*) and need factors (*e.g., routine check-up*) with adherence to surveillance mammography guidelines, while adjusting for potential confounder variables listed in Table 2. (e.g., survivor years, age, health insurance, employment and marital status) [4, 37, 38]. Predisposing factor, race-residence was significantly related with surveillance mammography adherence. Black women living in metropolitan /suburban counties had a 3.77 higher odds of adherence 95%CI (1.32–10.81) compared to White women living in metropolitan/suburban counties. However, a lower likelihood of adherence to surveillance mammography guidelines was found among Black women living in non-metropolitan counties compared to White women living in a non-metropolitan county (OR: 0.04; 95% CI: 0.00–0.50).

**Table 2:** Adjusted logistic regression results for adherence to surveillance mammography guidelines in non-Hispanic Black and non-Hispanic White Breast Cancer Survivors OR (95% Confidence Intervals CI)

	Adjusted OR	(95% CI)	*p*-value
**Predisposing Factors**			
Age			
< 50	Ref		
50–64	0.84	(0.30–2.37)	0.943
≥ 65	0.88	(0.30–2.54)	
Marital Status			
Married	1.10	(0.70–1.72)	
Not married	Ref		0.670
Race and Residence			
non-Hispanic Black Metropolitan	3.77	(1.32–10.81)	0.005*
non-Hispanic Black Non- Metropolitan	0.04	(0.00–0.51)	0.005*
non-Hispanic White Non- Metropolitan	1.04	(0.61–1.77)	
non-Hispanic White Metropolitan	Ref		
Perceived Health			
Very good	1.31	(0.62–2.78)	0.748
Good	1.17	(0.51–2.67)	
Poor	Ref		
Physical Health (M + SD)	1.00	(0.99–1.01)	0.797
Psychological Distress (M + SD)	1.00	(0.99–1.01)	0.527
**Enabling Factors**			
Health Insurance			
Yes	1.04	(0.17–6.19)	0.964
No	Ref		
Employment Status			
Employed	0.80	(0.44–1.44)	0.460
Non-employed	Ref		
Could not see doctor because of cost			
Yes	0.54	(0.23–1.25)	
No	Ref		0.152
Denied health insurance due to cancer			
Yes	0.95	(0.53–1.69)	0.865
No	Ref		
Have a personal doctor			
Yes	0.97	(0.46–2.06)	0.952
No	Ref		
**Need Factors**			
Survivor Years			
> 5 years from diagnosis	0.45	(0.28–0.71)	
≤ 5 years from diagnosis	Ref		< 0.001**
Received Follow-up Instructions			
Yes	1.09	(0.66–1.80)	
No	Ref		0.712
Length of time since last routine checkup			
Within 12 months	1.81	(0.94–3.51)	0.075
Within 2–5 years or 5 or more years	Ref		

Ref. denotes the reference subgroup of the variable.* *p*-value < .05, ** *p*-value < .01

^*^ *p* < 0.05; **p < 0.01

For enabling factors, having health care access was associated with adherence to surveillance mammography guidelines (*p* = 0.037); however, when adding survivor years (i.e., years from cancer diagnosis) to the final model health care access was no longer statistically significant.

Among need factors, women who were diagnosed more than 5 years ago had the lowest odds of adherence to surveillance mammography guidelines (OR:0.45;95%CI: 0.28–0.71). A marginal finding (i.e., *p*-value greater than 0.05 but less than 0.10) of meeting surveillance mammography was observed among survivors who had their last routine checkup within 12 months compared to, who had their last routine checkup within 2–5 years or more than 5 years (*p* = 0.075).

## Discussion

This study revealed potential explanations for racial disparities in the use of surveillance mammography guidelines, using 2016 BRFSS Data from non-Hispanic Black and non-Hispanic White BC survivors. In accordance with our conceptual framework, we found predisposing, enabling and need study variables associated with adherence in the use of surveillance mammography, in the last 12 months. Women diagnosed > 5 years had a lower likelihood of adherence compared to women diagnosed ≤ 5 years. While race was not independently associated with surveillance mammography, our results indicated that a combination of race and residential area were significantly related with adherence. Black survivors living in non-metropolitan residential counties were less likely to be adherent compared to White women living in non-metropolitan areas. This finding remained significant even after adjusting for potentially confounding factors (e.g., survivor years, health insurance, employment marital status).

Although adherence to surveillance mammography was less common among Black women living in non-metropolitan counties, their adherence rates were higher when living in metropolitan/suburban counties. These data provide insight for the role of socioeconomic disparities on racial differences in surveillance mammography behaviors, regarding area-level socioeconomic status (predisposing factor). In support of these findings, prior studies suggest when Black BC survivors live in lower-income residential areas suboptimal mammography adherence is followed [4, 16]. Similar results were found, in a different sample among survivors in a deprived class quintile based on their socioeconomic status index [23]. While these studies have comparable findings, they all have different socioeconomic measures and lack racial/ethnic diverse samples, which moderately show the influential role of socioeconomic status on Black women’s surveillance mammography behaviors. Much of what we know regarding the impact of socioeconomic status on cancer screening disparities are drawn from populations without cancer [41–43], more research is needed to uncover the complexity of socioeconomic disparities among BC survivors on surveillance behaviors, to inform future interventions. Targeted public health initiatives and interventions have successfully contributed to a historical increase in mammography screening rates among Black women without a history of BC [44–46]; yet initiatives targeting BC survivors are lacking. Future interventions should consider the environmental context and promote screening navigation programs among Black survivors living in non-metropolitan residential areas to support surveillance mammography behaviors.

Longer time elapsed from breast cancer diagnosis was a significant need predictor of lower adherence, regardless of race. Survivors diagnosed ≤ 5 years were more adherent compared to women diagnosed > 5 years. This finding is consistent with Breslau et al. (2010) study that found higher rates of mammography adherence among short-term survivors using the same time point from diagnosis (≤ 5 years). Similarly, other works that measured different time points concluded that longer time from diagnosis or treatment was associated with lower rates of mammography adherence [47–51]. One potential reason for these findings may be differences in short term vs. long term survivor’s intentions to receive a mammogram. Short-term BC survivors were more likely to receive a mammogram due to breast cancer or follow-up problems, while long-term BC survivors received their mammogram for breast surveillance purposes [37]. Survivors may adhere to screening practices because of their physician recommendation and NCCN recommendation for enhanced surveillance check-ups during the first 5-years are [4, 52, 53].Consistent with this and prior studies, our bivariate weighted analyses found women who reported having a routine check-up visit within the last 12 months had higher rates of surveillance mammography [54, 55]. Our results along with prior research suggest the importance of clinical visits and time from diagnosis on surveillance mammography, however gaps remain in understanding contextual factors that may differ for short-term vs. long-term survivor’s. Future research should investigate the role of social determinants to compare short-term and long-term survivors’ surveillance behaviors.

### Strengths & limitations

This study expands current knowledge about Black-White differences in surveillance mammography adherence rates. Strengths in this examination include our approach and analysis. We utilized a conceptual framework that is widely used in healthcare utilization to guide selection of study variables to assess the relationship on adherence to surveillance mammography guidelines. We examined an interaction between race and metropolitan residential area, which helped us disentangle the complexity of social determinant factors on surveillance behaviors. Despite these strengths there are limitations to highlight. The cross-sectional study design cannot confirm causality from study independent variables on adherence. The BRFSS dataset did not include clinical characteristics (e.g., histology, or surgery type) or medical claims data. Survey responses were self-reported which may result in overestimated mammography use. While we restricted our sample to survivors who had completed treatment from the cancer survivorship questionnaire, we do not know if women had a disease relapse, thus their mammography reported may have been for diagnostic purposes. Our inability to determine women living in small, remote rural areas among those living in non-metropolitan areas. Lastly, the BRFSS sample included 91% White women and 70.7% of the women were ≥ 65 thus, results are not generalizable to all BC survivors.

## Conclusion

We found that Black women living in non-metropolitan areas had significantly lower rates of surveillance mammography compared to White women in non-metropolitan areas. We also observed that longer time elapsed from disease negatively influenced survivors’ surveillance behaviors, regardless of race. Given the clinical benefit of receiving annual surveillance mammography guidelines, non-adherence to these guidelines among BC survivors is a public health issue. Improving Black women’s surveillance practices may aid to help close this group high mortality and adverse morbidity outcomes from BC. Our findings provide future research opportunities to further address surveillance mammography behaviors among survivors living in non-metropolitan areas. Addressing racial disparities in receiving breast cancer surveillance guidelines will help to advance breast cancer survivorship care.

## Data Availability

The BRFSS research data is publicly available on CDC website.
